# Two Major Archaeal Pseudomurein Endoisopeptidases: PeiW and PeiP

**DOI:** 10.1155/2010/480492

**Published:** 2010-11-11

**Authors:** Ganesh Ram R. Visweswaran, Bauke W. Dijkstra, Jan Kok

**Affiliations:** ^1^Department of Molecular Genetics, GBB, University of Groningen, Kerklaan 30, 9751 NN Haren, The Netherlands; ^2^Laboratory of Biophysical Chemistry, GBB, University of Groningen, Nijenborgh 4, 9747 AG Groningen, The Netherlands

## Abstract

PeiW (UniProtKB Q7LYX0) and PeiP (UniProtKB Q77WJ4) are the two major pseudomurein endoisopeptidases (Pei) that are known to cleave pseudomurein cell-wall sacculi of the members of the methanogenic orders *Methanobacteriales* and *Methanopyrales*. Both enzymes, originating from prophages specific for some methanogenic archaeal species, hydrolyze the *ϵ*(Ala)-Lys bond of the peptide linker between adjacent pseudomurein layers. Because lysozyme is not able to cleave the pseudomurein cell wall, the enzymes are used in protoplast preparation and in DNA isolation from pseudomurein cell-wall-containing methanogens. Moreover, PeiW increases the probe permeability ratio and enables fluorescence in situ hybridization (FISH) and catalyzed reporter deposition (CARD-) FISH experiments to be performed on these methanogens.

## 1. Introduction

**Figure 1:**
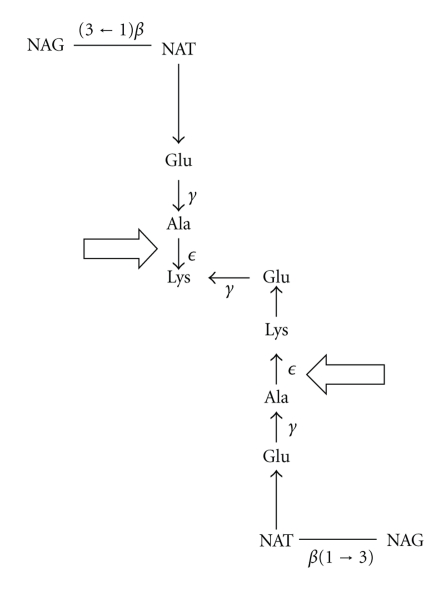
Dimer structure of pseudomurein and cleavage sites of pseudomurein endoisopeptidase (Pei). Block arrows indicate the cleavage site of Pei in the peptide subunit. The picture was adapted and modified from Kiener et al. [4].

Bacterial peptidoglycan hydrolases are among the most extensively studied hydrolases. Many three-dimensional structures of murein hydrolases have already been deposited in the Protein Data Bank; on the other hand, pseudomurein hydrolases are very poorly studied. Pseudomurein, the major cell wall component of gram-positive methanogenic archaea, is composed of *N*-acetyl-D-glucosamine (NAG) and *N*-acetyl-L-talosaminuronic acid (NAT) linked by a *β*(1 → 3)-glycosidic bond (see Figure 1) [1, 2]. Therefore, it is resistant to lysozyme and other bacterial hydrolases. The other cell wall polymers of methanogens include methanochondroitin and (glyco)protein surface (S)-layers [3]. Not much is known with regard to cell-wall-degrading lysins in methanogenic archaea. Pseudomurein endoisopeptidase (Pei) is the first enzyme known to hydrolyze the pseudomurein sacculi of archaeal methanogens [2, 4–7].

This short review not only gathers the available data on this unique enzyme but also enlightens its future research prospective. There is no three-dimensional structure of this enzyme despite its novelty and application potential. Further, research leading to the elucidation of the three-dimensional structure of pseudomurein endoisopeptidase may substantially increase our knowledge of its functionalities and possible applications to commercially and environmentally important methanogens.

## 2. The Pei Enzyme

Pseudomurein endoisopeptidase belongs to peptidase family C71, which contains peptidases that cleave the peptide subunits of pseudomurein sacculi (MEROPS database, http://merops.sanger.ac.uk/cgi-bin/famsum?family=C71). Pei is absent in most model organisms; so far, it has only been found in two prophages specific for some methanogens though two proteins from the archaeon *Methanobrevibacter smithii* may possess Pei activity (Interpro, http://www.ebi.ac.uk/interpro/IEntry?ac=IPR022119) [8]. The two prophage-derived Pei enzymes, PeiW (UniProtKB Q7LYX0) and PeiP (UniProtKB Q77WJ4) from *Methanothermobacter wolfeii * ΨM100 and *Methanothermobacter marburgensis* ΨM2, respectively, act as autolysins of the pseudomurein cell wall [9–11]. Both PeiW and PeiP cleave the oligopeptides that link the sugar chains of adjacent pseudomurein layers (see Figure 1) [4, 7]. The cleavage site is located between the *ϵ*-amino group of L-lysine and the carboxyl group of an L-alanine residue [4, 7]. As the hydrolysis of isopeptide bonds progresses, the sacculi disintegrate, facilitating further hydrolysis [4]. This distinct substrate specificity of the endoisopeptidases makes them part of a unique family of proteases.

PeiW and PeiP are the products of gene *orf28* of the prophages *M. wolfeii* ΨM100 and *M. marburgensis* ΨM2, respectively [6, 10]. PeiW contains 284 amino acid residues and has an apparent molecular weight of 33 kDa [4, 6]; on the other hand, PeiP is composed of 305 amino acids and has a predicted molecular mass (35.7 kDa) consistent with the observed mass of 36 kDa [12]. The stability and activity of PeiW and PeiP are influenced by both physical and chemical factors. His-tagged PeiW and PeiP showed a maximum activity at 71°C and 63°C, respectively [7]. No activity was observed at 37°C [4]. Both enzymes have a theoretical pI of around 9.4 (ExPASy) and were shown to be optimally active at a pH of 6.4 [7]. PeiW and PeiP are metal-activated peptidases as the metal chelator ethylenediaminetetraacetic acid (EDTA) inhibited both enzymes. Cell-wall-degrading activity can be restored by the addition of divalent cations like Ca^2+^ and Mg^2+^ [7].

## 3. Pei Structural Design

**Figure 2:**
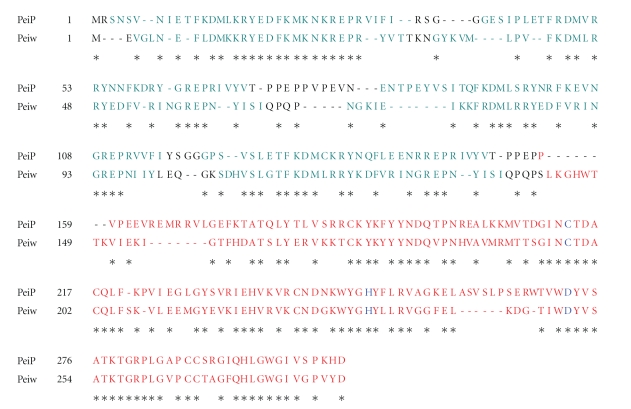
Protein sequence alignment of the pseudomurein endoisopeptidases PeiP and PeiW. The picture was generated with the SIM program of the ExPASy proteomics server (http://expasy.org/tools/sim-prot.html). N-terminal direct repeats in the pseudomurein binding repeat domain are shown in green, and the C-terminal catalytic domain is presented in red. The catalytic triad C-H-D is indicated in blue. ∗ indicates identical amino acid residues in PeiP and PeiW.

The domain organization of PeiP and PeiW indicates that they contain two distinct domains, an N-terminal pseudomurein binding repeat domain (PMBR) (pfam 09373) and a C-terminal catalytic cysteine protease domain (pfam 12386) (see Figure 2) [7, 11].

### 3.1. The N-Terminal Pseudomurein Binding Repeat Domain

The PMBR domains are not only present in PeiW and PeiP but also in 24 archaeal and 7 bacterial proteins (Interpro, http://www.ebi.ac.uk/interpro/IEntry?ac=IPR018975). The PMBR domains of PeiW and PeiP contain four direct repeats, each of which contains 30 to 35 amino acid residues (see Figure 2) (http://www.ebi.ac.uk/interpro/ISpy?ipr=IPR022119&tax=35237) [8]. As deletion of the PMBR domain in PeiW resulted in loss of binding of the enzyme to the pseudomurein layer, the domain is apparently involved in the binding of the enzyme to pseudomurein [11]. By deleting 1, 2, or 3 repeats from the PMBR domain of the surface (S)-layer protein MTH719 of *Methanothermobacter thermautotrophicus,* we could show that binding of the domain to pseudomurein only takes place if a minimum of three repeats are present [13]. The repeat structure of the binding domain is consistent with the polymeric nature of the substrate and probably serves as a determinant of substrate specificity [7]. The molecular function of the PMBR domain can thus be compared to that of the major murein binding domain, LysM, which is commonly found in cell wall hydrolases of bacteria and in other proteins [14, 15]. Steenbakkers et al. [11] have shown that PeiW hydrolyzes the pseudomurein layer by being associated with it, a process that is aided by the PMBR domain.

### 3.2. The C-Terminal Catalytic Domain

The catalytic endoisopeptidase domain of both PeiW and PeiP is involved in the cleavage of the linker peptide connecting the adjacent pseudomurein layers [6, 10]. When this domain was removed from PeiW or PeiP, the truncated enzymes did not lyse *Methanothermobacter* sp. cells [11]. The catalytic domain possesses a catalytic triad consisting of a cysteinyl residue, a histidyl residue, and an aspartyl residue (C-H-D) (see Figure 2) [7]. As the catalytic triad has a cysteinyl residue as putative nucleophile, PeiW and PeiP are highly sensitive to oxidative agents [7]. As discussed by Pfister and coworkers [10], PeiP catalyzes the *in vitro* lysis of *Methanothermobacter thermautotrophicus *Marburg cells only under anaerobic and reducing conditions [7, 10]. The catalytic triad C-H-D is homologous to that in animal transglutaminases like human blood clotting factor XIII and thiol proteases such as papain [7, 16].

## 4. Homologies

BLAST [17] searches revealed that PeiW and PeiP are very closely related to each other. An amino acid sequence comparison showed that the two enzymes have 53.4% identical amino acid residues and that the regions surrounding the catalytic triad residues are especially well conserved (see Figure 2) [6]. As mentioned earlier, the PeiW-related protein (UniProtKB A5UNW8) and a putative uncharacterized protein (UniProtKB D2ZMY6), from two different strains of *M. smithii*, do not have an N-terminal PMBR domain, but they do carry a C-terminal domain in which the amino acids surrounding the catalytic triad C-H-D are well conserved [8].

## 5. Applications of Pei

Lack of permeability of whole cells for oligonucleotide probes is always a major hurdle for the in situ detection of methanogens of the orders *Methanobacteriales* and *Methanopyrales* [2]. The probe hybridization ratio can be dramatically increased by treatment of cells with Pei, allowing for a very efficient analysis of methanogens by fluorescence in situ hybridization (FISH) experiments [2]. Pei treatment of whole cells is employed as a novel permeabilization method for catalyzed reporter deposition-(CARD-) FISH experiments on methanogens [9]. Pei is also widely used in the large-scale purification of undegraded genomic and plasmid DNA from *M. thermautotrophicus *and for the preparation of protoplasts from methanogens containing a pseudomurein cell envelope [4].
